# Case Report: Anti-N-Methyl-D-Aspartate Receptor Encephalitis in an Elderly Patient With Primary Sjögren's Syndrome

**DOI:** 10.3389/fneur.2021.656024

**Published:** 2021-05-20

**Authors:** Xia Li, Rui Kong, Qiuju Liao, Jing Ye, Yi Zhao

**Affiliations:** ^1^Department of Rheumatology and Allergy, Xuanwu Hospital, Capital Medical University, Beijing, China; ^2^Department of Neurology, Xuanwu Hospital, Capital Medical University, Beijing, China

**Keywords:** primary Sjögren's syndrome, autoimmune encephalitis, anti-N-methyl-D- aspartate receptor, cognitive dysfunction, Alzheimer's disease

## Abstract

Neurological manifestations of primary Sjögren's syndrome (SS) are diverse involving the peripheral and central nervous system. Anti-N-methyl-D-aspartate receptor (NMDAR) encephalitis, as the most prevalent autoimmune encephalitis, was rarely reported to be complicated with primary SS. Herein, we present an elderly patient with a 15-year history of primary SS presenting with progressive cognitive dysfunction due to anti-NMDAR encephalitis that was once misdiagnosed as primary degenerative dementia. Early recognition of anti-NMDAR encephalitis and initiation of treatment with steroids and immunosuppressant gained a favorable outcome. Our findings enhance the awareness that autoimmune encephalitis should be taken into account in the patients with primary SS presenting with progressive cognitive impairment.

## Introduction

Primary Sjögren's syndrome (SS) is a chronic systemic autoimmune disease characterized by lymphocytic infiltration of exocrine glands resulting in dry mouth and dry eyes (1). Many organs other than the exocrine glands may be affected including the skin, joints, lungs, gastrointestinal tract, pancreas, liver, and the kidneys. In addition to the extraglandular involvement, the patients with primary SS may exhibit a variety of peripheral neuropathies and/or central nervous system (CNS) manifestations. Neurological manifestations may be the first clinical manifestation in 25–60% of patients with primary SS and precede the diagnosis of primary SS on an average of 2 years (2). Cognitive impairment has been reported to be one of the common CNS involvement. Tezcan et al. reported that 39.2% of primary SS patients had moderate or severe cognitive dysfunction unrelated to the anti-N-methyl-D-aspartate receptor (NMDAR) antibody, anti-ribosomal-p and anti-ganglioside antibodies (3). However, autoimmune encephalitis, consisting of a wide variety of pathologic processes associated with the presence of antibodies against neuronal surface proteins, neuronal intracellular proteins, synaptic receptors (4), was rarely reported in primary SS. Anti-NMDAR encephalitis is the most prevalent autoimmune encephalitis involving anti-NMDAR antibodies, which is specific to the NR1 subunit of the NMDAR, and predominates in young women and children despite affecting people of any age (4). Herein, we report an elderly patient with a 15-year history of primary SS presenting with progressive cognitive dysfunction due to anti-NMDAR encephalitis.

## Case Presentation

**Figure 1:**
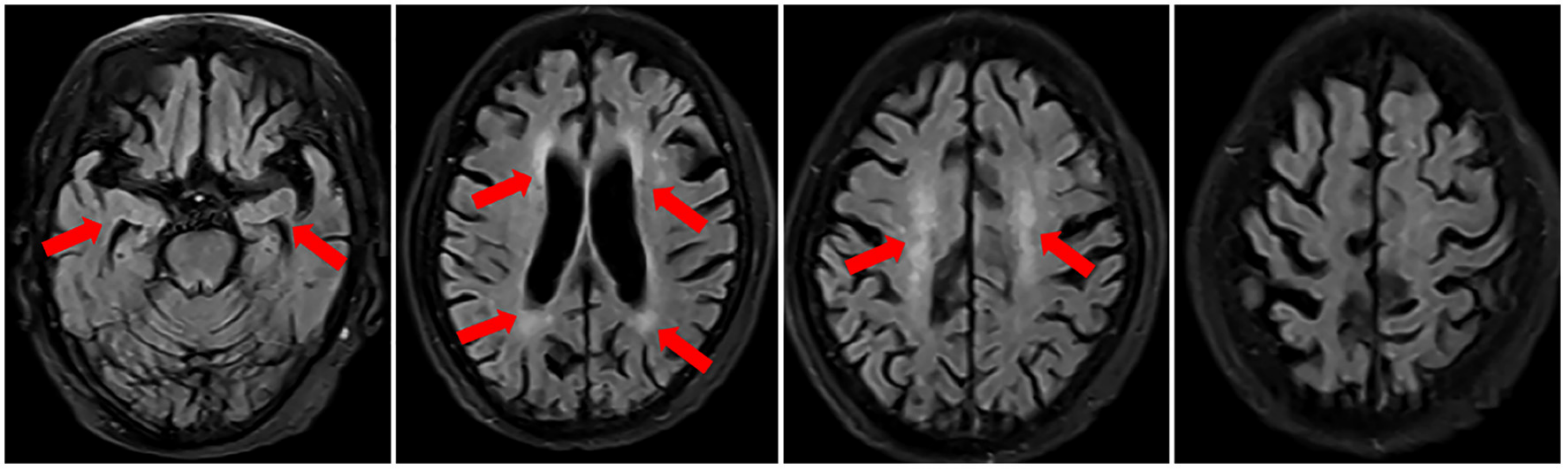
Brain MRI in November 2018 revealed multiple ischemic lesions, white matter degeneration and brain atrophy, including hippocampal atrophy.

A 76-year-old woman presented with a 4-year history of progressive muscle weakness of lower limbs. At the early stage, she still could walk without any aids. One year ago, a brain magnetic resonance image (MRI) scan revealed multiple ischemic lesions, white matter degeneration and encephalatrophy, including hippocampal atrophy (Figure 1). Her muscle weakness was getting worse gradually, and incontinence, dysarthria and dysphagia developed. One month before admission, she began to experience a decline in cognitive function and short-term memory deficit. Additionally, she was diagnosed with primary SS 15 years ago and treated with prednisone for 1 year. She denied any history of hypertension, diabetes and heart diseases.

**Figure 2:**
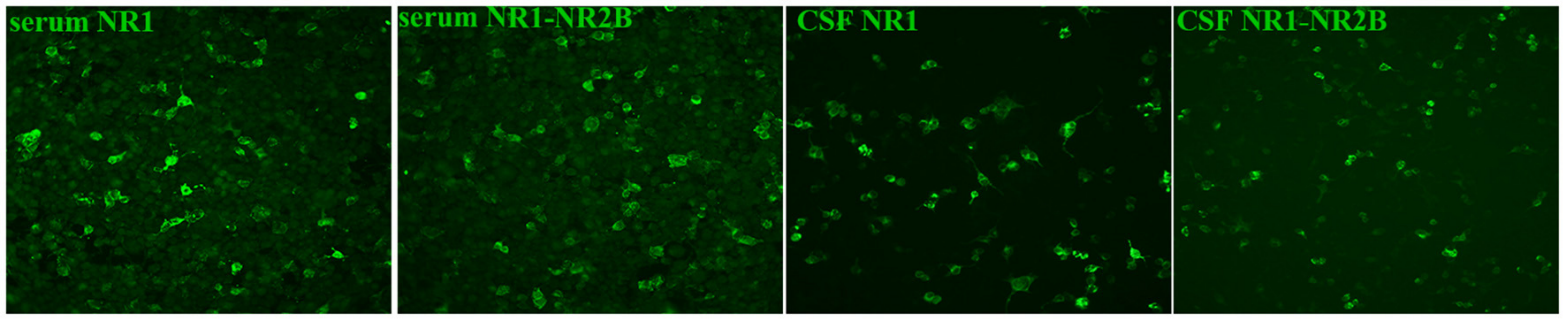
Human embryonic kidney 293 cells staining transfected with NR1 and NR1-NR2B subunits by using indirect immunofluorescence. As shown in this figure, the results were positive (200×).

**Figure 3:**
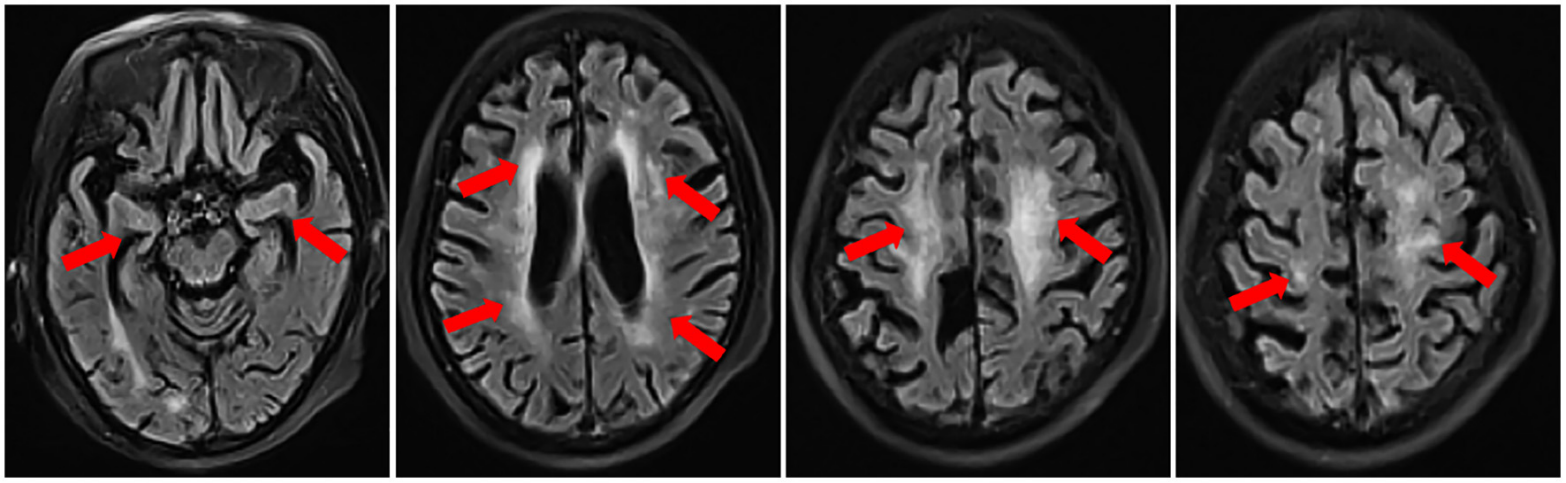
Brain MRI in September 2019 before treatment showed new scattered foci of T2- FLAIR hyperintensity within the periventricular, deep and subcortical white matter of the frontoparietal lobe and progressive brain atrophy, especially in the hippocampus.

The patient was first admitted to the Department of Neurology in September 2019. On admission, she had decreased consciousness, barylalia, and disorientation. Muscle strength of lower limbs was grade II. Cognitive dysfunction was confirmed through the tests of Mini-Mental State Examination, Montreal Cognitive Assessment and clock drawing. Laboratory tests were as follows: peripheral white blood cell (WBC) was 7.8 × 10^9^/L (4–10). Urinalysis, renal and liver functions were normal. Thyroid examination revealed she had Hashimoto's thyroiditis. Cerebrospinal fluid (CSF) analysis revealed 2 leukocytes, elevated protein level of 0.95 g/L (0.15~0.45) and normal glucose. The oligoclonal band (OB) was present in CSF. Transfection of human embryonic kidney (HEK) 293 cells with NR1 subunit or NR1-NR2B subunits of NMDAR was performed to test for the presence of NMDAR antibody (Figure 2) and it was confirmed by using a commercial kit (Euroimmune, Germany) with both cell-based assay (CBA) and rat brain immunostaining. Results showed that the anti-NMDAR was positive with a titer of 1:100 in both serum and CSF, and anti-aquaporin (AQP) 4 immunoglobulin (Ig) G antibody was weakly positive in serum. Pathogen detection was negative in the CSF. High-resolution computed tomography scan revealed interstitial lung disease. Brain MRI showed new scattered foci of T2-weighted-fluid-attenuated inversion recovery (FLAIR) hyperintensity within the periventricular, deep and subcortical white matter of the frontoparietal lobe without post-contrast enhancement (Figure 3). A primary diagnosis of Alzheimer's disease was made according to the elderly age of onset, a decline in cognitive function and hippocampal atrophy. Thus, she was transferred to the Department of Neurological Rehabilitation.

**Figure 4:**
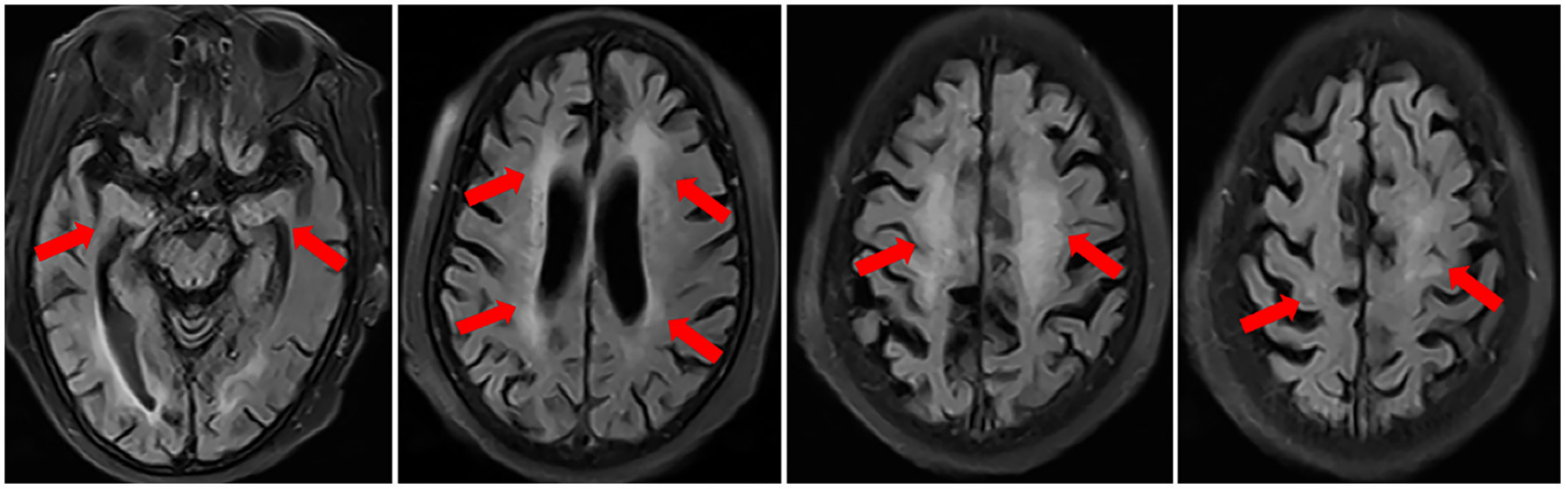
Repeated brain MRI after 2 months of treatment in November 2019 showed the scattered foci of T2-FLAIR hyperintensity disappeared partially.

Then a consultation of the rheumatologist was done owing to a previous diagnosis of primary SS. The patient had decay of teeth and recurrent swelling of parotid glands besides sicca symptoms. Immunologic examinations showed that IgG was 18.7 g/L (7.51~15.6), IgA 5.61 g/L (0.82~4.53); Complement C3 and C4 normal. ANA panel showed that ANA was positive with a titer of 1:320 (speckled pattern), anti-SSA positive (+++), anti-Ro-52 positive (+++), anti-double-strand DNA, anti-ribonucleoprotein, anti-Smith and anti-nucleosome antibody were all negative. Salivary scintigraphy showing delayed uptake and reduced excretion of tracer. Biopsy of the labial gland revealed focal lymphocytic sialadenitis. As a result, the diagnosis of primary SS complicated with anti-NMDAR encephalitis was considered (5). The patient was given methylprednisolone pulse therapy (500 mg/d for 3 days), followed by oral prednisone 60 mg per day (1 mg/kg/d), combined with mycophenolate mofetil 1,000 mg per day. After 1 week of treatment, the patient could communicate fluently, remember the recent events. The symptoms of dysphagia and dysarthria were alleviated. Repeated brain MRI after 2 months of treatment showed scattered foci of T2-FLAIR hyperintensity disappeared partially (Figure 4). And peripheral WBC was 5.69 × 10^9^/L. The anti-NMDAR antibody was positive with a titer of 1:10 in serum. There was no CSF results since the patient refused a second lumbar puncture. The dosage of prednisone was tapered gradually and a dose of 10 mg per day was maintained. In a follow-up of 1 year, she achieved a significant improvement.

## Discussion

Neuropsychiatric (NP) events are commonly found in patients with systemic lupus erythematosus (SLE) and primary SS. Previous studies demonstrated that circulating autoantibodies to the NR2 subunit of NMDAR was associated with the cognitive dysfunction and psychiatric disease (6–8). However, anti-NMDAR encephalitis has been found to be closely related to the antibodies against NR1 subunit of NMDAR detected by CBA. Zhao et al. studied the coexistence of autoimmune encephalitis and other systemic autoimmune diseases (9). There was 1 out of 111 patients with anti-leucine-rich glioma-inactivated 1 (LGI1) encephalitis and 1 out of 52 patients with anti–gamma aminobutyric acid B receptor (GABA_B_R) encephalitis coexisting with primary SS, respectively, but none of the 307 patients with anti-NMDAR encephalitis was coexisting with primary SS. The correlation between anti-NMDAR encephalitis and primary SS is yet to be clarified.

This elderly patient we reported had a 15-year history of primary SS presenting with a 4-year history of progressive muscle weakness of lower limbs. Brain MRI demonstrated diffuse brain atrophy, especially in the hippocampus. She was initially considered to have primary degenerative dementia related to Alzheimer's disease (AD). Misdiagnosis of AD could lead to different therapeutic outcomes since the autoimmune-related cognitive dysfunction is often reversible. However, the patient's symptoms worsened 2 months before admission, she had a rapid decline in cognitive function and memory impairment. We found that diffuse brain atrophy were progressive in this patient by comparing her present brain MRI with that of 1 year before the onset of cognitive dysfunction. However, in addition to the brain atrophy, there were scattered foci of T2-FLAIR hyperintensity within the periventricular, deep and subcortical white matter of the frontoparietal lobe. These were new lesions. As for this patient, anti-NR1 subunit of NMDAR antibody was detected with a titer of 1:100 in both CSF and serum by using the technique of CBA and rat brain immunostaining. Thus, the diagnosis of anti-NMDAR encephalitis combined with primary SS was established eventually. Subsequent remarkable response to immunotherapy further confirmed the diagnosis. We think that the patient's encephalitis may start 2 months before admission. The chronic cognitive impairment in the previous years probably is due to other causes such as cerebral ischemia and brain atrophy.

Actually, anti-NMDAR encephalitis is the most prevalent autoimmune encephalitis which predominates in young women and children despite affecting people of any age (4). Up to 58% of affected young female patients have an ovarian teratoma (4). As for this patient, positron emission tomography/computed tomography (PET/CT) scanning of the whole body was done and no solid tumors were detected. Additionally, abnormal heavy or light chain and monoclonal brand were negative with the detection of serum protein electrophoresis and immunofixation electrophoresis. As a consequence, the paraneoplastic syndrome could be ruled out.

Interestingly, anti-AQP4 IgG antibody was weakly positive in serum but not in CSF. As we know, the water channel protein AQP4 is expressed in the foot-processes of astrocytes throughout the central nervous system, and is the target of anti-AQP4-IgG in neuromyelitis optica spectrum disorders (NMOSD). There has been evidence that anti-NMDAR encephalitis could coexist with NMOSD sequentially or simultaneously (10). However, the patient did not develop optic neuritis or long-segment myelitis or other core clinical characteristics according to the international consensus diagnostic criteria for NMOSD (11). So the question is whether anti-AQP4 IgG could appear in autoimmune encephalitis. In fact, Zhao et al. have reported that multiple autoantibodies could be present in the same patient with autoimmune encephalitis, including the coexistence of anti-NMDAR and anti-AQP4 antibodies (1/307) (9). A recent investigation showed that between 4 and 7.5% of patients with anti-NMDAR encephalitis had concurrent glial-antibodies or neuronal-surface antibodies (12).

Cognitive impairment is not unusual in primary SS. Tezcan et al. reported that 39.2% of primary SS patients had moderate or severe cognitive dysfunction (3). Consequently, in terms of progressive cognitive dysfunction, autoimmune encephalitis should be taken into account and latent tumors need to be screened. Anti-NMDAR encephalitis is a potentially lethal but treatable autoimmune disorder. For this reason, early recognition and initiation of treatment are of great significance to facilitate a favorable outcome. This unique case emphasizes the need for a suspicion of anti-NMDAR encephalitis when approaching a primary SS patient with a decline in cognitive function.

## Conclusion

Anti-NMDAR encephalitis can be a rare form of CNS involvement in primary SS patients. When patients with primary SS have progressive cognitive decline, anti-NMDAR encephalitis should not be out of consideration. Detection of autoimmune encephalitis-related autoantibodies is very useful for differential diagnosis. Earlier recognition and intervention are of great significance to achieve a favorable outcome.

## Data Availability Statement

The original contributions presented in the study are included in the article/supplementary material, further inquiries can be directed to the corresponding author/s.

## Ethics Statement

Ethical review and approval was not required for the study on human participants in accordance with the local legislation and institutional requirements. The patients/participants provided their written informed consent to participate in this study.

## Author Contributions

XL wrote the manuscript. RK, QJL, JY, and YZ critically revised the manuscript. All authors read and approved the submitted version.

## Conflict of Interest

The authors declare that the research was conducted in the absence of any commercial or financial relationships that could be construed as a potential conflict of interest.
